# Modulation of mGluR1 signaling improves Purkinje cell dysfunction associated with spinocerebellar ataxia 14

**DOI:** 10.1016/j.isci.2026.117976

**Published:** 2026-10-20

**Authors:** Zsófia Sziber, Shiqi Tang, Paula Torrents-Solé, Aleksandar Kovacevic, Josef P. Kapfhammer

**Affiliations:** 1 Department of Biomedicine, University of Basel, Basel, Switzerland

**Keywords:** spinocerebellar ataxia, Purkinje cell, dendritic spine, PKCγ, mGluR1

## Abstract

Spinocerebellar ataxia type 14 (SCA14) is caused by mutations in protein kinase C gamma (PKCγ) that lead to constitutive kinase activity and Purkinje cell dysfunction in the cerebellum. PKCγ acts downstream of metabotropic glutamate receptor 1 (mGluR1) signaling; however, it remains unclear whether alterations in this upstream pathway contribute to the cellular defects induced by mutant PKCγ. Here, we investigated the role of mGluR1 signaling in PKCγ-dependent pathology using organotypic cerebellar slice cultures from a mouse model of SCA14. Modulation of mGluR1 signaling partially improved dendritic abnormalities and dendritic spine phenotypes associated with constitutive PKCγ activation. In addition, inhibition of mGluR1 reduced phosphorylation of PKC substrates and restored the enrichment of scaffold proteins and their synaptic proximity to mGluR1. Together, our results demonstrate that mGluR1 signaling contributes to Purkinje cell abnormalities induced by constitutive PKCγ activity and that modulation of mGluR1 can improve disease-associated cellular phenotypes in SCA14.

## Highlights

• mGluR1 inhibition improves dendritic defects mediated by the PKCγ-A24E mutant
• Spine density is restored by mGluR1 blockade in PKCγ-A24E Purkinje cells
• PKCγ-A24E culture shows reduced Homer3, Shank2, and mGluR1 enrichment
• mGluR1 inhibition reduces PKCγ activity in PKCγ-A24E, but not in PKCγ-S361G culture

## Graphical abstract

**Figure:**
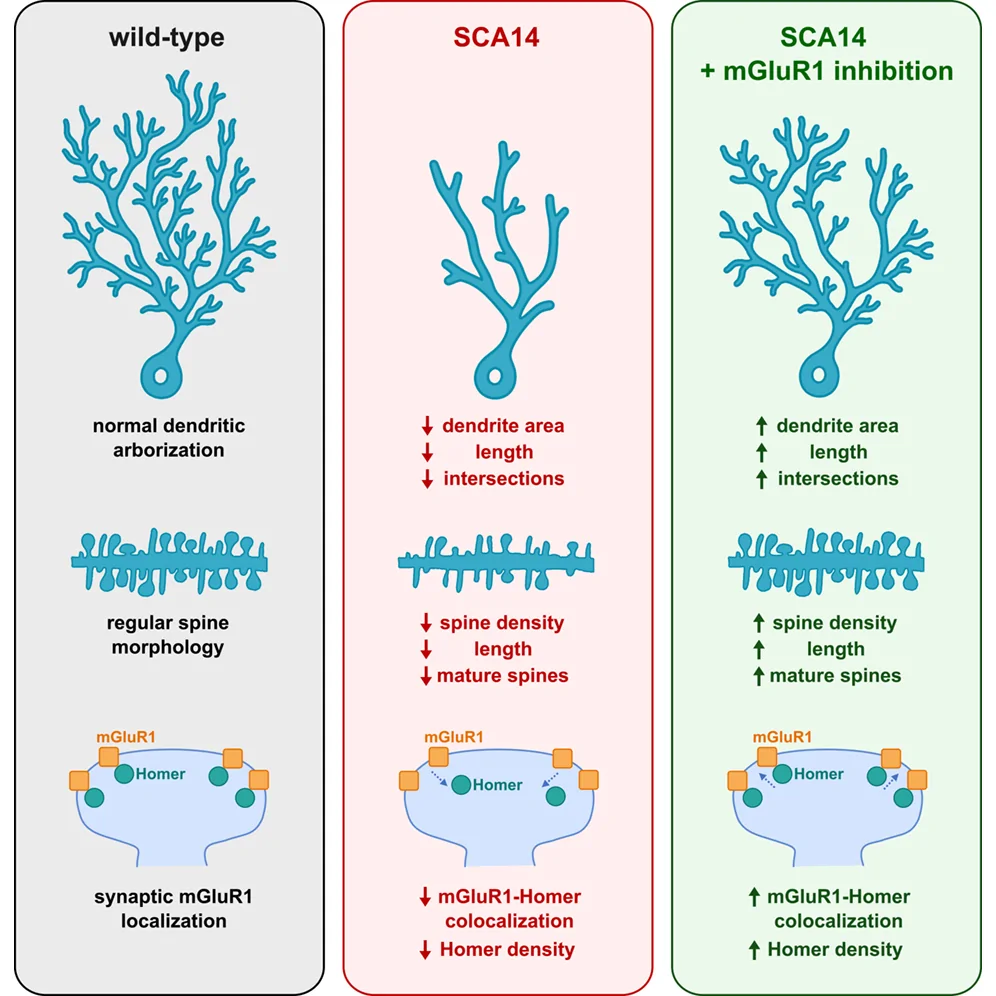

## Teaser

biological sciences; cell biology

## Introduction

Spinocerebellar ataxias (SCAs) are a genetically heterogeneous group of autosomal-dominant neurodegenerative disorders defined by progressive loss of motor coordination, cerebellar dysfunction, and degeneration of Purkinje cells in the cerebellar cortex. Patients commonly show gait instability, speech impairment, and poor limb coordination, caused by disrupted cerebellar processing of sensory and motor information.^1^ Although numerous SCA subtypes have been identified, the shared cerebellar phenotype suggests that common pathogenic mechanisms converge on Purkinje cell physiology and survival.^2^^,^^3^

Among these, spinocerebellar ataxia type 14 (SCA14) is caused by mutations in the *PRKCG* gene encoding protein kinase C gamma (PKCγ), a serine/threonine kinase highly expressed in cerebellar Purkinje cells. Mutations in PKCγ associated with SCA14 lead to altered kinase activity, disrupting normal cellular signaling pathways.^4^^,^^5^ These changes have been reported to reduce dendritic arborization and compromise the morphology of Purkinje cell dendrites.^6^^,^^7^

PKCγ is a conventional PKC isozyme that is tightly regulated and maintained in an autoinhibited state, in which regulatory domains prevent the kinase domain from phosphorylating substrates. Activation occurs upon binding of calcium and diacylglycerol (DAG), which release autoinhibition and allow the kinase to phosphorylate its targets.^8^^,^^9^ PKCγ plays a central role in multiple signaling pathways in the cerebellum. It regulates dendritic development, synaptic plasticity, cytoskeletal integrity, and the induction of long-term depression (LTD) at Purkinje cell synapses.^10^^,^^11^ Dysregulated PKCγ activity disrupts these processes and has been linked to abnormal Purkinje cell morphology, impaired synaptic functions, and motor deficits *in vivo*.^12^^,^^13^

Metabotropic glutamate receptor 1 (mGluR1) is another critical modulator of Purkinje cell function. It is highly expressed at perisynaptic sites of the dendritic spines in cerebellar Purkinje cells, and mediates glutamate-dependent signaling. Upon binding of glutamate, mGluR1 induces DAG production and calcium elevation, leading to PKCγ activation. Physiologically, mGluR1 regulates dendritic growth, synaptic plasticity, and intracellular calcium dynamics to support normal cerebellar function.^14^ In contrast, aberrant mGluR1 signaling has been reported in several forms of ataxia, with both reduced and enhanced activity depending on the disease model and stage. Evidence from mouse models and human studies suggests that mGluR1 plays a central role in cerebellar dysfunction, potentially involving downstream effectors such as PKCγ.^15^ Modulating the mGluR1-PKCγ signaling axis could therefore provide mechanistic insights and may represent a potential therapeutic approach for SCA.^16^^,^^17^

This study investigates how modulation of mGluR1 affects Purkinje cell function in mouse models of SCA14, a disorder characterized by overactive PKCγ.^1^ We addressed this question using pharmacological manipulations in organotypic cerebellar slice cultures derived from two distinct PKCγ mutant mouse lines that show SCA14-like phenotypes, with mutations located in different regions of the PKCγ protein. The PKCγ-A24E knockin model carries a point mutation in the pseudosubstrate domain, resulting in constitutive PKCγ activation,^7^ whereas the PKCγ-S361G transgenic line expresses a Purkinje cell-specific mutant transgene in the catalytic domain, leading to enhanced PKCγ kinase activity.^6^ Our aim is to investigate the mGluR1-PKCγ signaling pathway to better understand Purkinje cell dysfunction and to provide a basis for future studies on cerebellar impairments in SCA14.

## Results

### mGluR1 inhibition improves dendritic morphology compromised by the constitutively active PKCγ in the PKCγ-A24E mouse model

Dendritic morphology was analyzed in cultures from PKCγ-A24E wild-type and knockin mice with constitutively active PKCγ following modulation of mGluR1 signaling. In wild-type cultures, mGluR1 stimulation by 3,5-dihydroxyphenylglycine (DHPG) induced a strong reduction of the dendritic tree (Figures 1A–1D) consistent with previous findings,^18^ and a comparable decrease of the dendritic arbor was observed in Purkinje cells from the PKCγ-A24E knockin mouse line.^7^ In homozygous PKCγ-A24E cultures, treatment with the mGluR1 antagonist α-methyl-4-carboxyphenylglycine (MCPG) increased dendritic area (Figures 1A and 1B), total branch length (Figures 1A and 1C), and the number of intersections (Figures 1A and 1D), reversing dendritic deficits induced by increased PKCγ activity. In contrast, activation of mGluR1 with DHPG further reduced the dendritic area and branch length (Figures 1A–1C). These results indicate that inhibition of mGluR1 signaling improves dendritic architecture in Purkinje cells when PKCγ activity is constitutively elevated.

**Figure 1:**
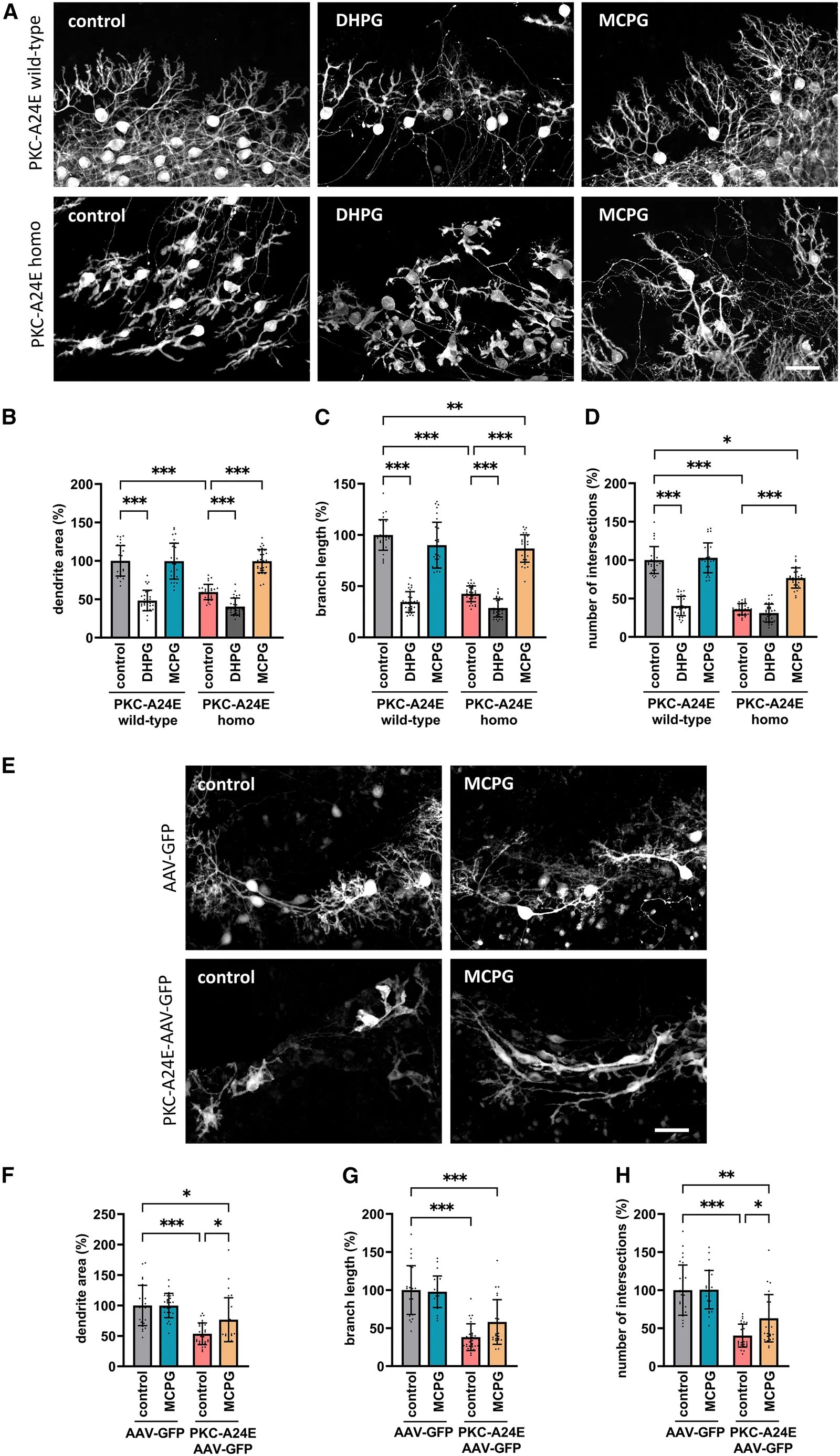
Analysis of dendritic morphology in PKCγ-A24E wild-type and homozygous cultures, as well as in cultures transduced with AAV-GFP or PKC-A24E-AAV-GFP, following modulation of mGluR1 signaling (A) Representative fluorescence images of calbindin-immunolabeled Purkinje cells after DHPG or MCPG treatment (scale bar, 50 μm). Quantification of (B) dendrite area, (C) branch length, and (D) number of intersections (% of control) in Purkinje cells. (E) Microscopy images of Purkinje cells transduced with AAV-GFP or PKC-A24E-AAV-GFP followed by MCPG treatment (scale bar, 50 μm). Analysis of (F) dendrite area, (G) branch length, and (H) number of intersections (% of control) in transduced Purkinje cells. Data were obtained from 3 independent cultures generated from 9 animals per genotype, with 30 cells analyzed per condition. Individual cells were considered biological replicates (*n* = 30; B–D). Data were collected from 3 independent cultures from 6 animals, with 27–29 cells analyzed per condition. Cells were treated as biological replicates (*n* = 27–29; F–H). Comparisons were made using Welch’s and Brown-Forsythe ANOVA followed by Dunnett’s post hoc test (B–D), or Kruskal-Wallis test followed by Dunn’s multiple comparison (F–H). All data are displayed as mean ± SD. ∗*p* < 0.05, ∗∗*p* < 0.01, ∗∗∗*p* < 0.001.

To further confirm the effects of mGluR1 inhibition on dendritic morphology, wild-type cerebellar slice cultures were transduced with adeno-associated viruses (AAVs) driving Purkinje cell-specific expression of GFP (AAV-GFP) or constitutively active PKCγ (PKC-A24E-AAV-GFP), and dendritic architecture was analyzed. In Purkinje cells transduced with PKC-A24E-AAV-GFP, we found that MCPG treatment improved dendrite morphology, including dendritic area (Figures 1E and 1F) and branching complexity (Figures 1E and 1H), while branch length showed only a slight, not significant increase (Figures 1E and 1G). Interestingly, the improvement was less pronounced in transduced cultures compared to homozygous PKCγ-A24E cultures. This difference may be due to variability in viral transduction efficiency and expression levels, which can influence cellular responses and thereby limit the degree of morphological rescue.

### MCPG does not reverse the morphological alterations of the dendrites in cultures from the PKCγ-S361G mouse model with increased kinase activity

Dendritic morphology was analyzed in Purkinje cells from PKCγ-S361G transgenic cultures, which express the catalytic domain mutant PKCγ-S361G construct specifically in Purkinje cells, leading to enhanced PKCγ activity.^6^ In this model, transgene expression can be suppressed by tetracycline through the Tet-Off system, therefore tetracycline-treated cultures served as a wild-type condition. As expected, dendritic area, branch length, and complexity were higher in tetracycline-treated wild-type cultures compared to mutant conditions (Figures 2A–2D). DHPG treatment reduced these parameters in control slices but did not further decrease dendritic area in mutant cultures (Figures 2A and 2B). According to our results, no significant differences were observed between untreated and MCPG-treated Purkinje cells in PKCγ-S361G mutant cultures. MCPG treatment did not affect dendritic area, total branch length, or the number of intersections, and overall dendritic complexity remained unchanged (Figures 2A–2D), indicating that dendritic morphology in PKCγ-S361G cultures is not influenced by mGluR1 inhibition.

**Figure 2:**
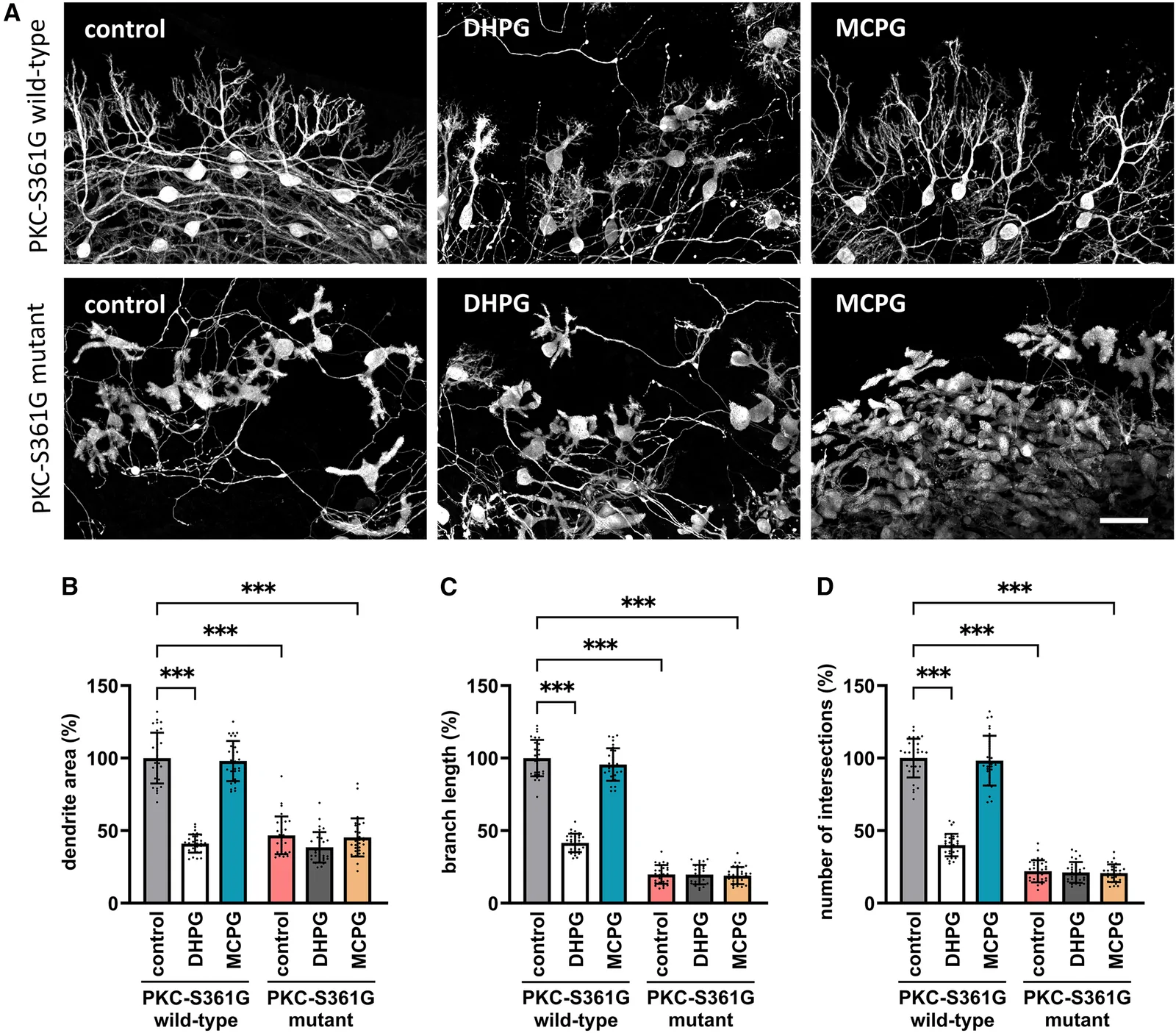
Investigation of dendritic morphology in PKCγ-S361G wild-type and mutant cultures, in response to mGluR1 modulation (A) Calbindin-labeled Purkinje cells after DHPG or MCPG treatment (scale bar, 50 μm). Quantification of (B) dendrite area, (C) branch length, and (D) number of intersections (% of control). Data were obtained from 3 independent cultures generated from 9 animals, with 30 cells analyzed per condition. Cells were considered biological replicates (*n* = 30). Comparisons were made using Welch’s and Brown-Forsythe ANOVA followed by Dunnett’s post hoc test. All data are displayed as mean ± SD. ∗∗∗*p* < 0.001.

### Spine density and morphology are restored by mGluR1 inhibition in Purkinje cells expressing constitutively active PKCγ in the PKCγ-A24E mouse model

The morphological parameters of dendritic spines were analyzed in PKCγ-A24E wild-type and homozygous cerebellar slice cultures after pharmacological manipulation of mGluR1. Compared to wild-type controls, constitutive PKCγ activity was associated with reduced spine density, shorter protrusion length, and decreased spine head width (Figures 3A–3D), consistent with previous observations.^19^ A comparable decrease in spine morphology was observed in wild-type Purkinje cells upon DHPG exposure. Treatment with the mGluR1 antagonist MCPG increased spine density and restored protrusion length and head width in homozygous cultures (Figures 3A–3D). Spine subtype analysis showed a decreased proportion of mushroom-type spines in a constitutive PKCγ background, which was increased by MCPG treatment. Furthermore, our results indicate a reduction in the number of immature filopodia in the PKCγ-A24E background in response to MCPG addition (Figures 3A and 3E), suggesting that dendritic spine alterations are modulated by the upstream regulator of PKCγ, mGlur1.

**Figure 3:**
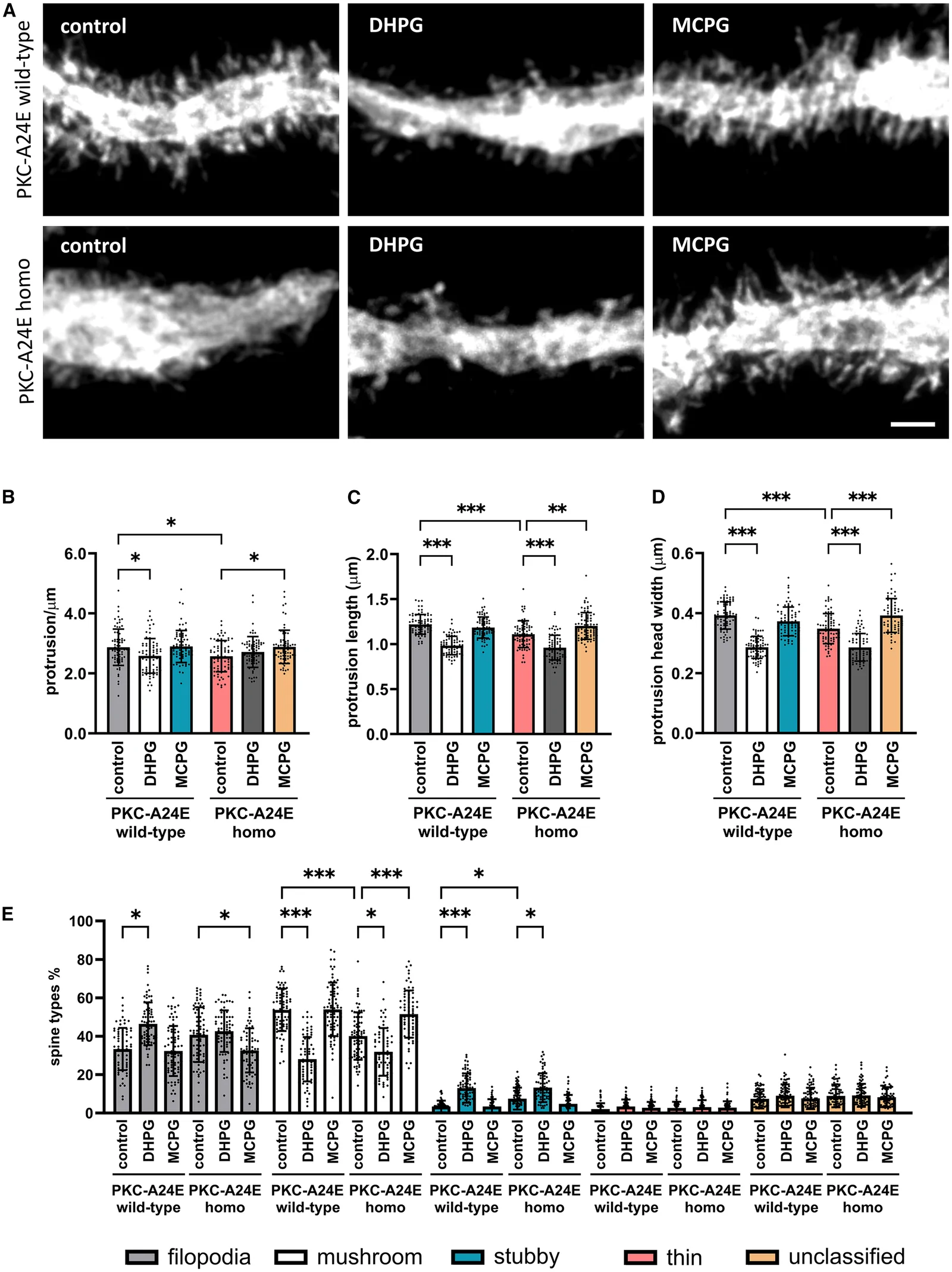
Analysis of dendritic spine density, length, and morphology in organotypic slice cultures of PKCγ-A24E wild-type and homozygous mouse lines (A) Representative microscopy images of dendrites of calbindin-labeled Purkinje neurons treated with DHPG or MCPG (scale bar, 2 μm). (B) Mean protrusion density, (C) length and (D) head width of dendritic spines of Purkinje cells. (E) Mean percentage of filamentous, mushroom, stubby, thin or unclassified spines. Data were collected from 3 independent cultures generated from 7 animals per genotype, with 15 cells and 75 dendritic branches analyzed per condition. Dendritic branches were treated as biological replicates (*n* = 75). Data were compared using one-way ANOVA with Bonferroni correction (C) or Welch’s and Brown-Forsythe ANOVA followed by Games-Howell post hoc test (D) or Kruskal-Wallis test followed by Dunn’s multiple comparison test (B and E). All data are displayed as mean ± SD. ∗*p* < 0.05, ∗∗*p* < 0.01, ∗∗∗*p* < 0.001.

### mGluR1 inhibition restores Shank2 enrichment and Shank2-mGluR1 colocalization in PKCγ-A24E cultures

Following spine analysis, we examined whether synapse number and size were altered using Shank2 as a postsynaptic marker protein. Consistent with the spine data, both the number and area of Shank2-positive puncta were reduced in PKCγ-A24E homozygous cultures compared to wild-type controls (Figures 4A–4C). Furthermore, DHPG treatment decreased Shank2 enrichment and the size of Shank2-positive areas in wild-type Purkinje cells, whereas treatment with the mGluR1 antagonist MCPG restored the number and area of Shank2-positive postsynaptic puncta in homozygous cultures (Figures 4A–4C). We next investigated the spatial relationship between Shank2 and mGluR1. Overall, Shank2 and mGluR1 showed a high degree of colocalization, which was reduced in homozygous cells and restored following MCPG treatment (Figures 4A and 4D). These findings suggest that constitutive PKCγ activation is associated with reduced synapse number and altered postsynaptic organization, and that mGluR1 inhibition can restore synaptic architecture under these conditions.

**Figure 4:**
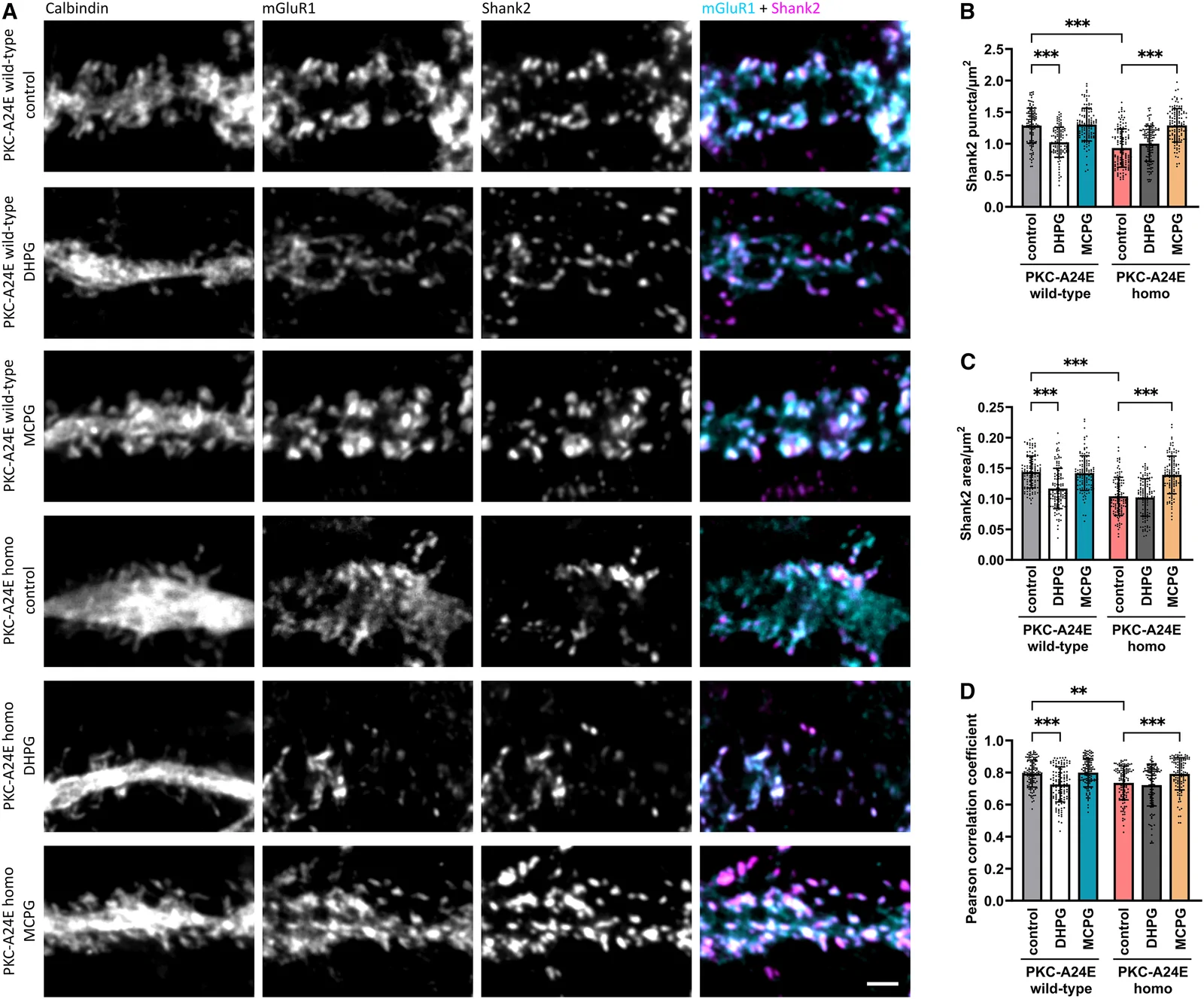
Quantification of Shank2 puncta number, area, and colocalization with mGluR1 in dendrites of Purkinje cells obtained from PKCγ-A24E wild-type and homozygous mice (A) Purkinje cell dendrites were immunolabeled for mGluR1 and Shank2 after DHPG or MCPG treatment (scale bar, 2 μm). Overlays indicate the colocalization between mGluR1 (cyan) and Shank2 (magenta). Quantification of (B) Shank2 puncta number, (C) Shank2 area per unit dendrite area, and (D) Pearson’s correlation coefficient for mGluR1 and Shank2 colocalization within dendrites. Data were obtained from 3 independent cultures derived from 8 animals per genotype, with 24 cells and 120 dendritic branches analyzed per condition. Dendritic branches were treated as biological replicates (*n* = 120). Comparisons were made using Welch’s and Brown-Forsythe ANOVA followed by Games-Howell post hoc test (B), or Kruskal-Wallis test followed by Dunn’s multiple comparison (C and D). All data are displayed as mean ± SD. ∗∗*p* < 0.01, ∗∗∗*p* < 0.001.

### Homer3 enrichment and mGluR1 colocalization are reduced in Purkinje cells from PKCγ-A24E cultures

Following the analysis of Shank2, we examined whether the enrichment and synaptic organization of Homer3, a postsynaptic density scaffolding protein and direct binding partner of mGluR1, are altered in homozygous PKCγ-A24E cultures and upon mGluR1 inhibition. DHPG treatment led to a decrease in both the number and size of Homer3 puncta in wild-type Purkinje cells (Figures 5A–5C). In homozygous PKCγ-A24E cultures, Homer3 enrichment was reduced (Figures 5A and 5B), accompanied by a decrease in the size of Homer3-positive puncta (Figures 5A and 5C) and reduced colocalization with mGluR1 (Figures 5A and 5D). Treatment with the mGluR1 antagonist MCPG restored Homer3 enrichment and puncta size and increased its colocalization with mGluR1 (Figures 5A–5D). These findings indicate that constitutive PKCγ activation disrupts synaptic scaffold organization, consistent with the effects observed for Shank2, and that inhibition of mGluR1 can reverse these alterations in an overactive PKCγ background.

**Figure 5:**
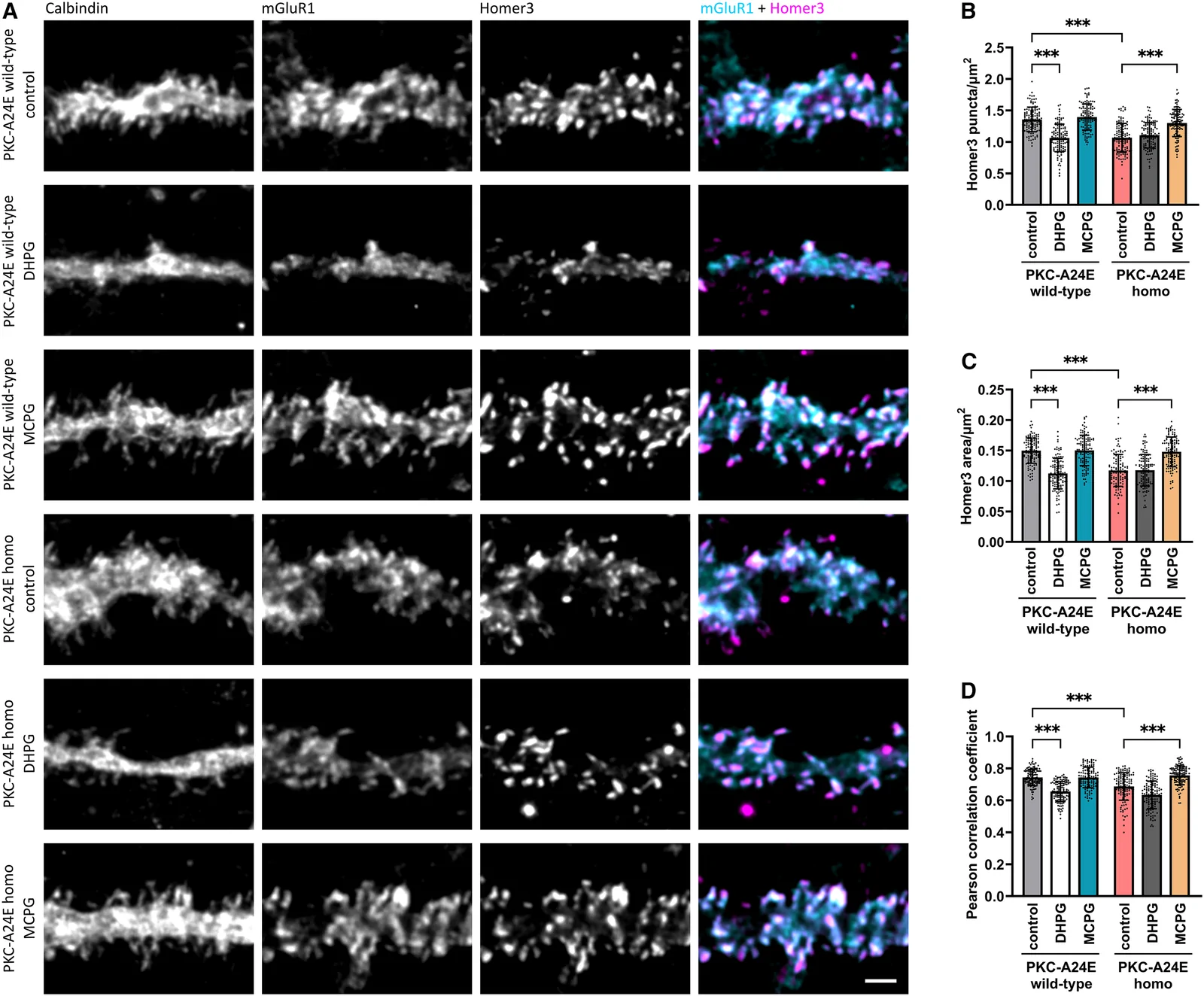
Analysis of Homer3 puncta density, size, and colocalization with mGluR1 in dendrites of Purkinje cells from PKCγ-A24E wild-type and homozygous mice (A) Purkinje cell dendrites were immunostained for mGluR1 and Homer3 following DHPG or MCPG treatment (scale bar, 2 μm). Colocalization of mGluR1 (cyan) and Homer3 (magenta) is shown in the merged images. Quantification of (B) Homer3 puncta number, (C) Homer3 area, and (D) Pearson’s correlation coefficient for mGluR1 and Homer3 colocalization within dendrites. Data were collected from 3 independent cultures generated from 9 animals per genotype, with 24 cells and 120 dendritic branches analyzed per condition. Dendritic branches were considered biological replicates (*n* = 120). Comparisons were made using Welch’s and Brown-Forsythe ANOVA followed by Games-Howell test (B and C), or Kruskal-Wallis test followed by Dunn’s multiple comparison (D). All data are displayed as mean ± SD. ∗∗∗*p* < 0.001.

### MCPG enhances mGluR1-PKCγ colocalization in PKCγ-A24E Purkinje cell dendrites

Next, we examined whether dendritic enrichment of mGluR1 and PKCγ was altered across experimental conditions. Overall, no major differences in dendritic mGluR1 or PKCγ enrichment were detected between groups, with the exception of DHPG treatment (Figures 6A–6C). As expected, PKCγ signal intensity was reduced in homozygous cultures (Figures 6A and 6C), consistent with previous observations.^7^ Interestingly, a significant increase in PKCγ intensity was observed in homozygous cultures following MCPG treatment. We then investigated the spatial relationship between mGluR1 and PKCγ by colocalization analysis. Colocalization was reduced in homozygous cultures, likely reflecting the decreased availability of PKCγ. Notably, MCPG treatment produced an increase in mGluR1-PKCγ colocalization in homozygous neurons (Figures 6A and 6D). This suggests that mGluR1 inhibition partially restores the spatial coupling of mGluR1 and PKCγ within dendritic compartments.

**Figure 6:**
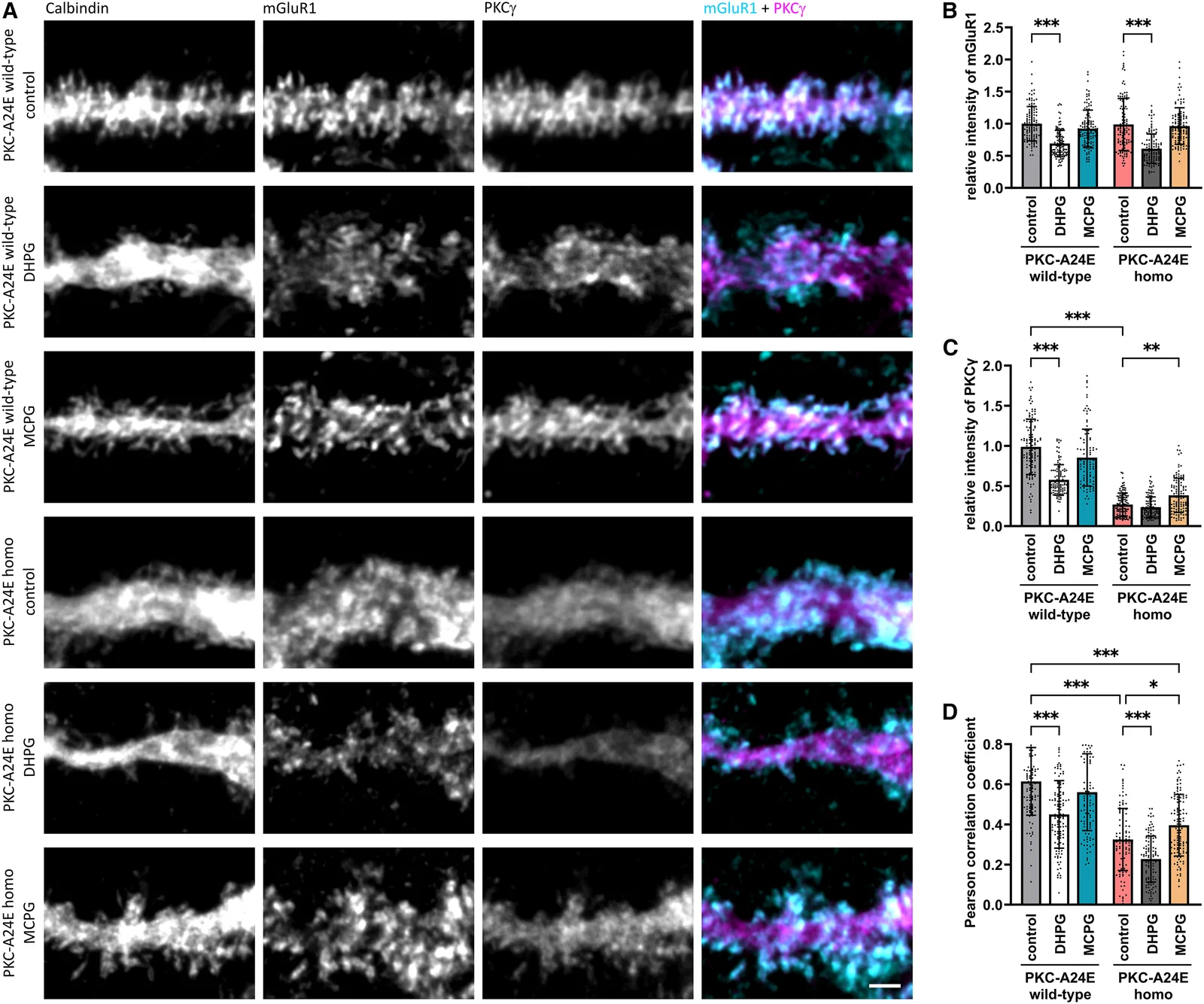
Analysis of mGluR1 and PKCγ intensity and colocalization in Purkinje neuron dendrites (A) Representative fluorescence microscopy images of mGluR1 and PKCγ immunostaining in dendrites of calbindin-positive Purkinje neurons treated with DHPG or MCPG. Colocalization of mGluR1 (cyan) and PKCγ (magenta) is shown in the merged images. Images were obtained from organotypic cerebellar slice cultures of PKCγ-A24E wild-type and PKCγ-A24E homozygous mouse lines (scale bar, 2 μm). Quantification of (B) mGluR1 and (C) PKCγ signal intensity, and (D) Pearson’s correlation coefficient between mGluR1 and PKCγ within Purkinje cell dendrites. Data were obtained from 3 independent cultures derived from 9 animals per genotype, with 24 cells and 120 dendritic branches analyzed per condition. Dendritic branches were treated as biological replicates (*n* = 120). Data were compared using Kruskal-Wallis test followed by Dunn’s multiple comparison test. All data are displayed as mean ± SD. ∗*p* < 0.05, ∗∗*p* < 0.01, ∗∗∗*p* < 0.001.

### GluR2 enrichment is decreased and fewer GluR2-containing AMPA receptors are present at the postsynapse in PKCγ-A24E cerebellar slices

To clarify the functional consequences of the molecular and structural alterations observed in Purkinje cells, we analyzed GluR2 subunit of α-amino-3-hydroxy-5-methylisoxazole-4-propionic acid (AMPA) receptor enrichment and its colocalization with the postsynaptic scaffold protein Shank2 in Purkinje cell dendrites. We found that GluR2 intensity was reduced in DHPG-treated conditions compared to untreated wild-type cultures (Figures 7A and 7B). In addition, DHPG treatment decreased the colocalization of GluR2 with the postsynaptic scaffold protein Shank2, suggesting fewer GluR2-containing AMPA receptors at postsynaptic sites (Figures 7A and 7C). Notably, PKCγ-A24E Purkinje neurons showed reduced signal intensity and colocalization between AMPA receptors and Shank2, indicating decreased abundance and synaptic localization of GluR2 compared to wild-type controls. In contrast, MCPG treatment restored GluR2 enrichment and colocalization with Shank2 (Figures 7A–7C), suggesting that constitutively active PKCγ leads to impaired postsynaptic AMPA receptor enrichment and localization that is reversed by mGluR1 inhibition.

**Figure 7:**
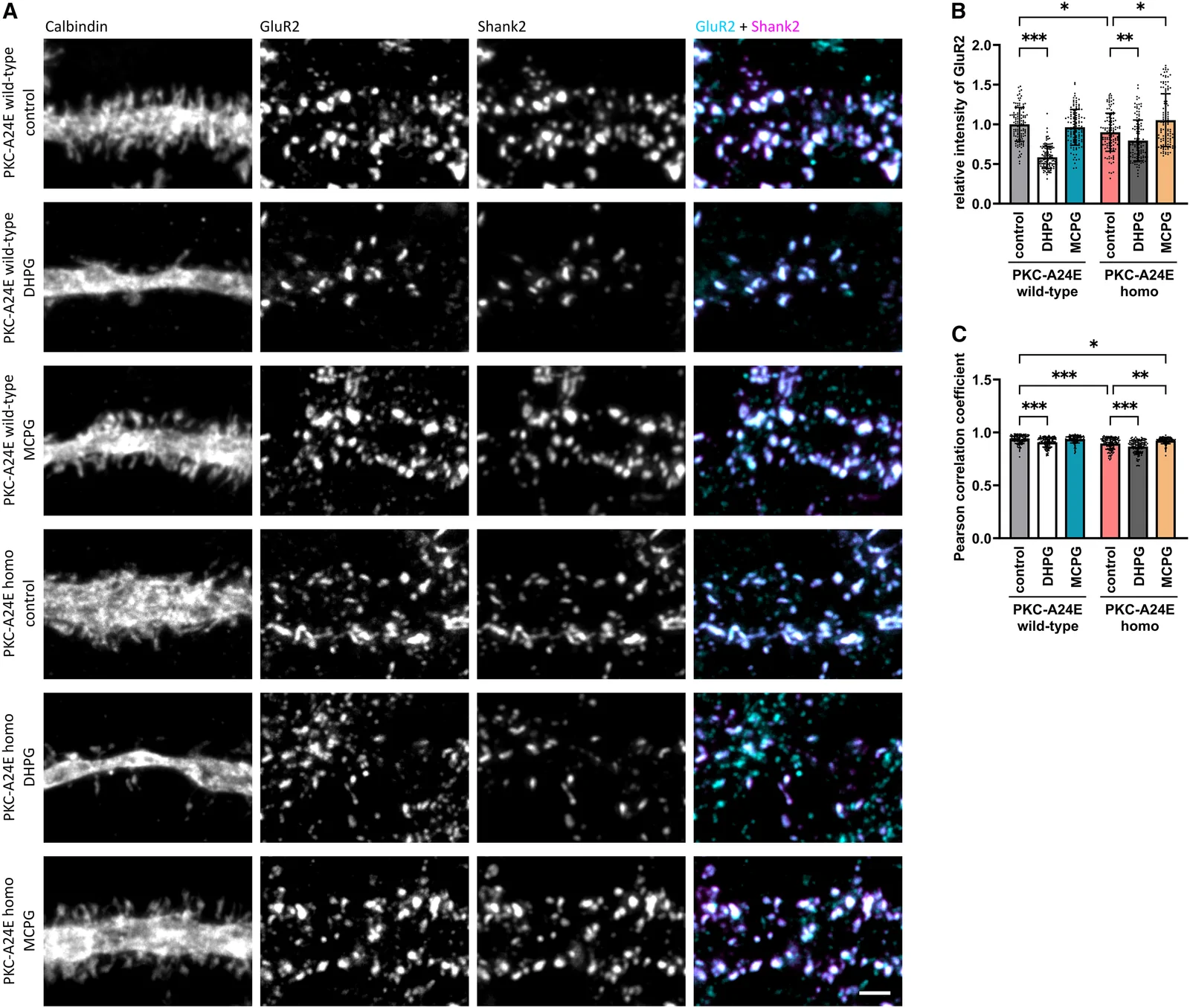
Quantification of GluR2 intensity and colocalization with Shank2 in Purkinje cell dendrites (A) Purkinje cell dendrites were immunostained for GluR2 and Shank2 following DHPG or MCPG treatment (scale bar, 2 μm). Colocalization of GluR2 (cyan) and Shank2 (magenta) is shown in the merged images. Images were obtained from organotypic cerebellar slice cultures of PKCγ-A24E wild-type and PKCγ-A24E homozygous mouse lines. Quantification of (B) GluR2 intensity, and (C) Pearson’s correlation coefficient for GluR2 and Shank2 colocalization within Purkinje neuron dendrites. Data were obtained from 3 independent cultures derived from 7 animals per genotype, with 24 cells and 120 dendritic branches analyzed per condition. Dendritic branches were considered biological replicates (*n* = 120). Data were compared using Kruskal-Wallis test followed by Dunn’s multiple comparison test. All data are displayed as mean ± SD. ∗*p* < 0.05, ∗∗*p* < 0.01, ∗∗∗*p* < 0.001.

### mGluR1 inhibition reduces PKCγ activity in PKCγ-A24E cultures, but not in PKCγ-S361G

PKCγ activity was investigated using a general phosphoserine (pSer) PKC substrate antibody to determine whether PKC substrate phosphorylation was altered in PKCγ-A24E and PKCγ-S361G cultures in response to mGluR1 inhibition. Consistent with previous observations, phosphorylation was increased in PKCγ-A24E homozygous cultures.^7^ Treatment with MCPG reduced the level of phosphorylated PKC substrates compared to untreated samples (Figures 8A and 8B), indicating that PKCγ activity is decreased when mGluR1 is inhibited. A slight increase in total PKCγ protein levels was observed in wild-type slices treated with MCPG, whereas homozygous cultures showed a marked reduction in PKCγ expression (Figures 8A and 8C), as reported previously.^7^ In mutant PKCγ-S361G cultures, enhanced PKC substrate phosphorylation was measured,^20^ accompanied by elevated PKCγ expression (Figures 8D–8F). This is consistent with an earlier study reporting that two-week old mice have increased PKCγ expression, which declines with age.^6^ In PKCγ-S361G mutant cultures, PKC substrate phosphorylation was not affected by MCPG treatment (Figures 8D and 8E), indicating that PKCγ-S361G mutation does not respond to mGluR1 modulation, which is in line with the absence of dendritic morphological changes in these cultures.

**Figure 8:**
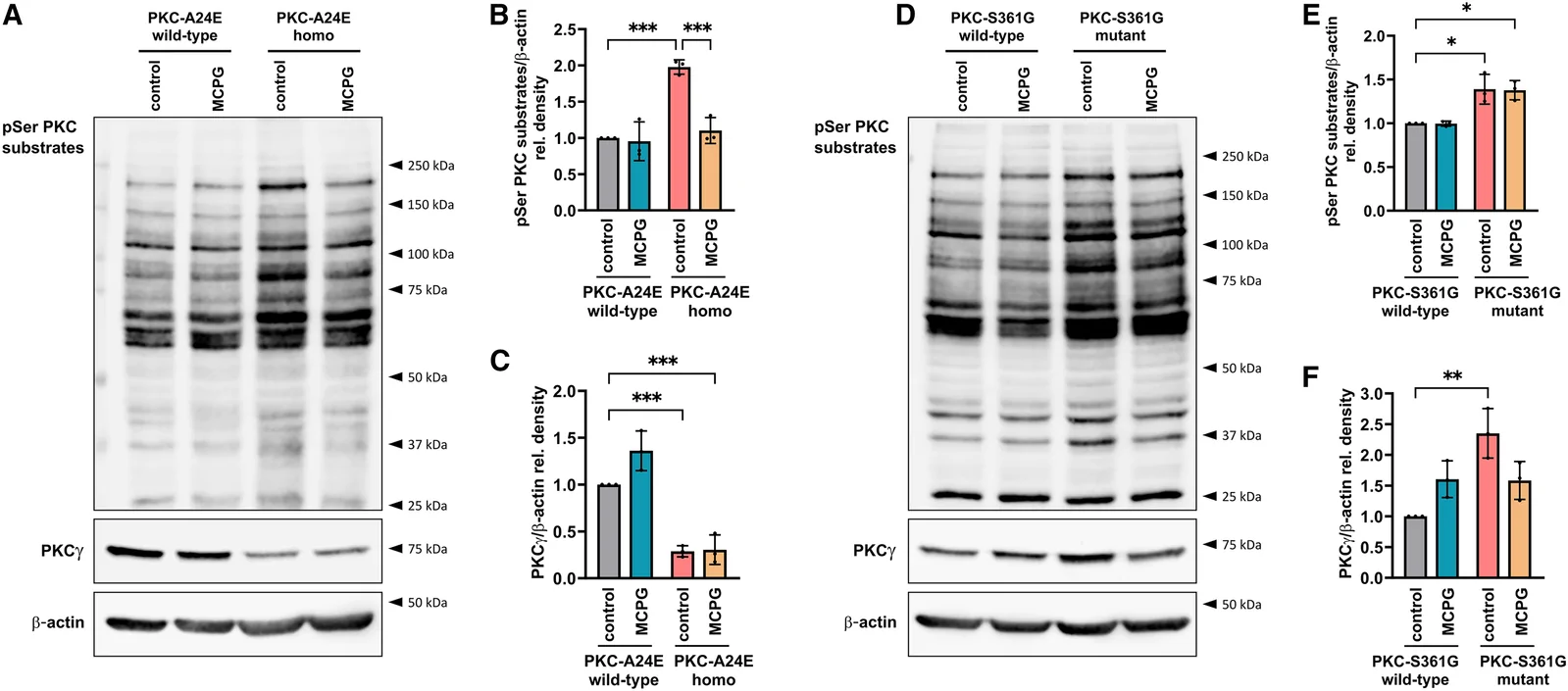
Analysis of PKCγ kinase activity in PKCγ-A24E and PKCγ-S361G cultures Representative western blot images of phosphorylated PKC substrates and total PKCγ protein in organotypic cerebellar slices from (A) PKCγ-A24E and (D) PKCγ-S361G cultures. β-Actin served as a loading control. Quantification of (B and E) pSer PKC substrate level and (C and F) PKCγ signal normalized to β-actin and to wild-type controls. Data were obtained from 3 independent cultures generated from 9 animals per genotype. The 3 independent cultures were treated as biological replicates (*n* = 3). Data were compared using one-way ANOVA with Bonferroni correction. All data are displayed as mean ± SD. ∗*p* < 0.05, ∗∗*p* < 0.01, ∗∗∗*p* < 0.001.

## Discussion

In this study, we investigated how mGluR1 signaling influences PKCγ-dependent regulation of Purkinje cell morphology in mouse models of SCA14. We found that pharmacological inhibition of mGluR1 with MCPG partially rescued the dendritic phenotype, improved synaptic organization, and reduced phosphorylation of PKC substrates in the PKCγ-A24E mouse model. Modulation of mGluR1 affected changes induced by constitutive active PKCγ, suggesting that upstream receptor activity contributes to dysfunctional PKCγ signaling in cerebellar Purkinje cells. In contrast, inhibition of mGluR1 had no effect in the PKCγ-S361G mouse model with increased kinase activity.

It has been reported that activation of mGluR1 induced by DHPG leads to a strong reduction in the size of the Purkinje cell dendritic tree.^18^ Based on previous findings, elevated PKCγ activity similarly disrupts the dendritic arbor,^21^ and the A24E mutant of PKCγ causes a comparable phenotype in cerebellar Purkinje cells.^7^ These observations suggest that mGluR1 activation and PKCγ overactivity converge on the same pathway, leading to similar dendritic defects in Purkinje cells. Based on our results, in slice cultures from the PKCγ-A24E mutant, the mGluR1 inhibitor was able to partially rescue the compromised dendrites caused by the mutation. This indicates that dendritic alterations in the PKCγ-A24E mutant remain sensitive to upstream mGluR1 signaling, which normally provides calcium and DAG required for PKCγ activation. In contrast, mGluR1 inhibition did not rescue dendritic morphology in PKCγ-S361G cultures, suggesting that this mutant does not require calcium and DAG to maintain its effect.

The improvement observed at the level of dendritic arborization in PKCγ-A24E slice cultures was also seen at the level of dendritic spines. MCPG treatment increased spine density and shifted spine composition toward more mature mushroom type spines, while reducing the proportion of filopodia in PKCγ-A24E background. It has been shown that PKCγ regulates dendritic spine morphology and development in Purkinje cells. Activation of PKCγ decreases spine density and the number of mature spines.^19^^,^^22^ mGluR1 has also been shown to contribute to the regulation of synaptic strength and to stabilize dendritic protrusions.^23^ Furthermore, a previous study reported a functional connection between mGluR1 and PKCγ, indicating that mGluR1 activation increases PKCγ-dependent phosphorylation of calcium/calmodulin-dependent protein kinase II β (CaMKIIβ), which modifies F-actin bundling and therefore plays a role in regulating spinogenesis and spine stabilization.^24^

The cerebellar Purkinje cell synapse has been widely used as a model system for investigating synapse elimination in the developing central nervous system. Purkinje cells are initially innervated by multiple climbing fibers, which are subsequently refined to a single persistent dendritic input. Previous studies have demonstrated that signaling through the mGluR1-PKCγ pathway is essential for the late phase of climbing fiber synapse elimination, as disruption of this cascade or genetic deletion of its molecular components impairs the removal of synapses during postnatal development.^17^^,^^25^ These observations highlight that both mGluR1 and PKCγ are critically involved in synapse elimination during Purkinje cell development, in addition to their role in spinogenesis.

The observed decrease in spine density in homozygous PKCγ-A24E Purkinje cells was accompanied by reduced expression of the postsynaptic scaffold protein Shank2, an indirect binding partner of mGluR1 that helps stabilize it at the synapse.^26^ MCPG treatment restored Shank2 levels in PKCγ-A24E cultures, and the spatial association between Shank2 and mGluR1 was improved. A previous study has reported that Shank2 knockout mice show reduced excitatory synapse density in cerebellar Purkinje cells and impaired motor coordination,^27^ suggesting that Shank2 is critical for maintaining synapse stability and proper spine organization in Purkinje cells. Furthermore, phosphorylation of Shank proteins is important for their synaptic enrichment, where they play a crucial role in cross-linking synaptic proteins in a signaling complex.^26^^,^^28^

Homer3, a direct binding partner of mGluR1, is highly enriched at the postsynapse of cerebellar Purkinje cells.^29^^,^^30^ Similar to Shank proteins, Homers undergo activity-dependent regulation of their postsynaptic localization, thereby modulating scaffolded receptors such as mGluR1.^31^^,^^32^ It has been demonstrated that CaMKII-dependent phosphorylation of Homer3 reduces its interaction with target molecules^33^ and modulates calcium signaling induced by mGluR1 activation in Purkinje cells.^34^ We observed a decrease in colocalization between Homer3 and mGluR1 in homozygous cultures, which was improved following MCPG treatment. This suggests that alterations in the phosphorylation state of Homer3 lead to its dissociation from mGluR1, resulting in a more diffuse distribution of mGluR1 within the membrane rather than being properly organized at synaptic sites. Such a mislocalization may contribute to increased or dysregulated receptor activity. In our experimental model, inhibition of mGluR1 activity by MCPG at the membrane may prevent excessive calcium signaling and subsequent Homer3 phosphorylation, thereby promoting the association between Homer3 and mGluR1. This is supported by a previous study on mGluR5, which showed that activity-dependent CaMKII phosphorylation of Homer disrupts receptor-scaffold interactions, leading to hyperexcitability and abnormal mGluR5 signaling in cortical and hippocampal cultures.^35^ Mislocalization of mGluR1 at Purkinje cell dendritic spines has been reported in a mouse model of SCA5, demonstrating that altered receptor distribution might underlie cerebellar dysfunction.^36^

At the parallel fiber-Purkinje cell synapse, glutamate release activates AMPA receptors and initiates synaptic changes. During long-term potentiation (LTP), increased synaptic expression and activity of AMPA receptors enhances synaptic transmission, whereas LTD is associated with AMPA receptor internalization and reduced synaptic efficacy. Glutamate release also activates mGluR1, which increases calcium and DAG concentration, leading to activation of PKC signaling pathways and subsequent AMPA receptor endocytosis, a key mechanism underlying LTD.^37^ It has been reported that DHPG induces mGluR1 desensitization^38^ and LTD in cerebellar Purkinje neurons,^39^ which is in line with our observation that AMPA receptor enrichment is reduced in dendrites and that colocalization with the postsynaptic scaffold protein Shank2 is decreased in DHPG-treated cultures. Such changes are characteristic of AMPA receptor internalization during LTD and indicate reduced synaptic efficacy, ultimately leading to synaptic weakening.^40^ Our results show that constitutive PKCγ activity contributes to reduced abundance and postsynaptic localization of GluR2-containing AMPA receptors in Purkinje cell dendrites, which may lead to decreased synaptic efficacy and is potentially modulated by upstream mGluR1 signaling.

The reductions in spine density, AMPA receptors and changes in postsynaptic scaffold protein distribution associated with increased PKCγ activity may reflect either pathological alterations or homeostatic mechanisms. In this view, spine loss and remodeling of postsynaptic structures could represent a compensatory response that helps stabilize synaptic input and maintain neuronal function by adjusting receptor distribution and cellular excitability.^41^ Dell'Orco et al. demonstrated in a mouse model of SCA1 that structural changes in Purkinje cells serve as an adaptive response rather than a purely degenerative process. They showed that neuronal shrinkage compensates for altered membrane excitability by restoring ion channel density and maintaining normal firing properties.^42^ Another study demonstrates that mGluR1 mediates homeostatic regulation of intrinsic excitability in cerebellar Purkinje cells, with constitutive activation possibly leading to reduced intrinsic excitability.^43^ These findings suggest that neurons engage protective or compensatory mechanisms to minimize harmful effects under suboptimal conditions.

It has been shown that expression of SCA14-related mutant PKCγ increases the amplitude of mGluR1-mediated slow excitatory postsynaptic potentials, indicating enhanced mGluR1-dependent synaptic responses.^12^ Such increased activity may disrupt the spatial organization of PKCγ and mGluR1 in dendrites. Based on our results, we observed a slight improvement in PKCγ colocalization with mGluR1 in PKCγ-A24E slice cultures following MCPG treatment without affecting their dendritic abundance, suggesting that restoration of function might occur through improved spatial association rather than altered protein amount. We found that overall PKCγ expression levels were decreased in PKCγ-A24E cerebellar slices, whereas PKCγ catalytic activity was increased, as expected and previously reported.^7^

Comparison of the two PKCγ mutations investigated in this study revealed several interesting differences. Phosphorylation of PKC substrates was reduced by MCPG in PKCγ-A24E cultures, whereas phosphorylation in PKCγ-S361G cerebellar slices remained unchanged. This aligns with our dendritic morphology results, where MCPG restored dendrites in the PKCγ-A24E background but had no effect in PKCγ-S361G, highlighting the functional differences between the two mutations. The PKCγ-A24E variant appears to remain sensitive to upstream modulation, whereas the catalytic domain PKCγ-S361G mutation drives stronger, sustained activity and is possibly unresponsive. The pseudosubstrate domain mutant PKCγ-A24E increases catalytic activity indirectly through reduced or absent autoinhibition of the kinase domain. Due to the conformational changes associated with PKCγ-A24E activation, the mutant protein undergoes subsequent enzyme degradation.^44^ These findings are also consistent with the observation that mutated PKCγ-A24E remains sensitive to PKC inhibitors such as GF 109203X or Gö6983, whereas PKCγ-S361G is unaffected by PKC inhibitor treatment. PKCγ-S361G induces a marked alteration in spine morphology compared to PKCγ-A24E, characterized by a higher proportion of mushroom and stubby spines.^19^ These differences in expression patterns, the mode of PKCγ activation and protein degradation may contribute to the more pronounced effects observed in the PKCγ-S361G model. Taken together, SCA14-associated PKCγ mutations converge on similar signaling pathways regulating dendritic arborization and PKCγ-dependent substrate phosphorylation, but differ in their dependence on upstream mGluR1 signaling and in their regulatory context, including differences in expression patterns. In summary, these findings highlight the possible role of mGluR1-dependent PKCγ signaling and substrate phosphorylation in maintaining dendritic structure.

It is well established that mGluR1 is involved in ataxia, with both loss- and gain-of-function contributing to disease phenotypes.^15^^,^^45^ Although no effective clinical interventions currently exist, preclinical studies suggest that positive and negative allosteric modulators of mGluR1 can restore ataxic behavior in mouse models, indicating that targeting mGluR1 could be a viable therapeutic strategy.^46^

PKCγ is a major downstream effector of mGluR1, driving SCA14-related pathology and causing calcium homeostasis dysfunction,^4^ presumably through mGluR1 activity.^16^ PKCγ may contribute to SCA14 through its downstream substrates, which in turn modulate mGluR1 function, such as RGS8^20^ or CaMKII.^24^ Our findings suggest that mGluR1 is not simply an upstream regulator of PKCγ, as mGluR1 appears to modulate some PKCγ mutants, while mutated PKCγ can, in turn, influence mGluR1 activity. Furthermore, the relationship between mGluR1 and PKCγ in ataxia remains largely unexplored. Our study particularly focused on the PKCγ-A24E knockin model, in which the mutation is located within the regulatory domain, where most SCA14-associated mutations are found.^5^ We show that inhibition of mGluR1 activity reduced PKCγ activity in the PKCγ-A24E mutant slice cultures, thereby partially rescuing compromised dendritic branching and decreasing phosphorylation of PKC substrates, both of which are commonly associated with SCA14. Importantly, the PKCγ-A24E mutant remains sensitive to mGluR1 inhibition, indicating that upstream mGluR1 signaling can still modulate mutant PKCγ activity. Although PKCγ activity is constitutively increased in this mutant, it is not completely independent of upstream mGluR1 activity. Furthermore, we demonstrate that this mutant affects not only dendritic morphology but also dendritic spine organization, AMPA localization and postsynaptic architecture, suggesting a broader impact of SCA14-linked PKCγ dysfunction on synaptic structure. Together, these findings extend current models of mGluR1-PKCγ signaling by linking mutation-specific signaling properties to structural synaptic alterations in SCA14. In summary, our study suggests that modulating mGluR1 activity might improve SCA14-related deficits in mouse models, highlighting a potential pathway that future studies could explore in more detail for therapeutic interventions.

### Limitations of the study

This study has several limitations that should be considered when interpreting the results. First, while we provide evidence for altered mGluR1-PKCγ signaling in a PKCγ-A24E mouse model, we did not directly measure functional synaptic output using electrophysiological recordings. Such experiments would further strengthen the mechanistic link between molecular changes and synaptic function. Second, while our data support a functional link between mGluR1 signaling and PKCγ-dependent phenotypes, we did not dissect the downstream signaling events linking mGluR1 activation to PKCγ activity. Therefore it remains unclear how mGluR1-derived second messengers, such as calcium and DAG, contribute to the maintenance or regulation of the PKCγ-A24E mutant activity. Addressing this point with functional assays would further strengthen the mechanistic interpretation of the pathway. Finally, prolonged pharmacological stimulation with DHPG may induce secondary effects, including changes in cell viability and receptor trafficking, which may complicate interpretation of long-term signaling changes. In contrast, short-term DHPG treatment may be more suitable to assess acute effects and to distinguish them from secondary changes associated with chronic stimulation.

## Resource availability

### Lead contact

Requests for further information and resources should be directed to and will be fulfilled by the lead contact, Zsófia Sziber (zsofiailona.sziber@unibas.ch).

### Materials availability

All unique/stable reagents generated in this study are available from the lead contact without restriction.

### Data and code availability

• This paper does not report original code.
• All data generated in this study are included in the article.
• Any additional information required to reanalyze the data reported in this paper is available from the lead contact upon request.

## Acknowledgments

This research was supported by the Department of Biomedicine (DBM) Microscopy Core Facility, University of Basel animal facilities, and a Swiss National Science Foundation (SNSF) grant (grant number 310030-189083).

## Author contributions

Z.S., writing – original draft, writing – review and editing, supervision, methodology, conceptualization, validation, investigation, and formal analysis; S.T., validation, formal analysis, and investigation; P.T.-S., formal analysis; A.K., formal analysis; J.P.K., writing – review and editing, supervision, and funding acquisition.

## Declaration of interests

The authors declare no competing interests.

## STAR★Methods

### Key resources table

**Table:** 

REAGENT or RESOURCE	SOURCE	IDENTIFIER
**Antibodies**		

Mouse monoclonal anti-Calbindin	Swant	Cat# CB300PUR; RRID:AB_3542811
Guinea pig polyclonal anti-Calbindin	Synaptic Systems	Cat# 214 004; RRID:AB_10550535
Mouse monoclonal anti-PKC Gamma	Thermo Fisher Scientific	Cat# 13–3800; RRID:AB_2533015)
Guinea pig polyclonal anti-Shank2	Synaptic Systems	Cat# 162 204; RRID:AB_2619861
Mouse monoclonal anti-mGluR1	BD Biosciences	Cat# 556331; RRID:AB_396369
Rabbit monoclonal anti-mGluR1	Cell Signaling Technology	Cat# 12551; RRID:AB_2797953
Rabbit polyclonal anti-Homer3	Synaptic Systems	Cat# 160 303; RRID:AB_10804288
Mouse monoclonal anti-GluR2	Synaptic Systems	Cat# 182 411AT488; RRID:AB_2943616
Mouse monoclonal anti-β-actin	Sigma-Aldrich	Cat# A5441; RRID:AB_476744
Rabbit polyclonal anti-phospho-serine (pSer) PKC substrate	Cell Signaling Technology	Cat# 2261; RRID:AB_330310
Mouse monoclonal anti-PKC Gamma	Proteintech	Cat# 66429-1-Ig; RRID:AB_2881800
Goat anti-mouse Alexa Fluor 488	Thermo Fisher Scientific	Cat# A-11001; RRID:AB_2534069)
Goat anti-rabbit Alexa Fluor 568	Thermo Fisher Scientific	Cat# A-11011; RRID:AB_143157)
Goat anti-mouse Alexa Fluor 647	Abcam	Cat# ab150115; RRID:AB_2687948
Goat anti-guinea pig Alexa 488	Thermo Fisher Scientific	Cat# A-11073; RRID:AB_2534117)
Goat anti-guinea pig Alexa 647	Thermo Fisher Scientific	Cat# A-21450; RRID:AB_2535867
Donkey anti-mouse IRDye 800CW	LI-COR Biosciences	Cat# 926–32212; RRID:AB_621847
Donkey anti-rabbit IRDye 680LT	LI-COR Biosciences	Cat# 926–68023; RRID:AB_10706167

**Bacterial and virus strains**		

Stellar competent cells	Takara Bio	Cat# 636763
pAAV/L7-6-GFP-WPRE	PackGene Biotech AG	USXH240105498-1, Lot# 15549T
pAAV/L7-6-GFP-WPRE-PKC-A24E	PackGene Biotech AG	USXH240702520-1, Lot# 17509T

**Chemicals, peptides, and recombinant proteins**		

(RS)-3,5-dihydroxyphenylglycine (DHPG)	Tocris Bioscience	Cat# 0342
(S)-α-methyl-4-carboxyphenylglycine (MCPG)	Tocris Bioscience	Cat# 0337
Tetracycline hydrochloride (tetracycline)	PanReac AppliChem	Cat# A2228
ProLong Gold Antifade Mountant	Thermo Fisher Scientific	P36930

**Critical commercial assays**		

CloneAmp HiFi PCR Premix	Takara Bio	Cat# 639298
In-Fusion Snap Assembly Master Kit	Takara Bio	Cat# 638947

**Experimental models: Organisms/strains**		

Mouse: FVB/NRj	In house breeding	N/A
Mouse: PKCγ-A24E	Shimobayashi et al.^7^	N/A
Mouse: PKCγ-S361G	Ji et al.^6^	N/A

**Oligonucleotides**		

pAAV/L7-6-GFP-WPRE forward primer: 5′-TTATGCTAGCTTCTAGAGCATGGCTACGT-3′	This paper	N/A
pAAV/L7-6-GFP-WPRE reverse primer: 5′-ATAAACGCGTCTCGAGTCTAGAGCATGGC-3′	This paper	N/A
pAAV/L7-6-GFP-WPRE-PKC-A24E forward primer: 5′-CGTGAGGACTACCGGTATGGCTGGTCTGGGCCCC-3′	This paper	N/A
pAAV/L7-6-GFP-WPRE-PKC-A24E reverse primer: 5′-CATGGTGGCGTCGACCATGACGGGCACAGGCACTGG-3′	This paper	N/A

**Recombinant DNA**		

plasmid: pAAV/L7-6-GFP-WPRE (original)	Nitta et al.^47^	RRID:Addgene_126462
plasmid: PKC-A24E	Shimobayashi et al.^7^	N/A
plasmid: pAAV/L7-6-GFP-WPRE	This paper	N/A
plasmid: pAAV/L7-6-GFP-WPRE-PKC-A24E	This paper	N/A

**Software and algorithms**		

Image Studio Lite 5.2	LI-COR Biosciences	RRID:SCR_013715
Fiji	Fiji	RRID:SCR_002285
GraphPad Prism 10	GraphPad Software	RRID:SCR_002798

### Experimental model and study participant details

#### Mouse models and organotypic slice cultures

Animal experiments were carried out in accordance with the EU Directive 2010/63/EU for animal experiments and were reviewed and approved by Swiss authorities (National no. 38376, Cantonal no. 1708 04). Animal studies were conducted in compliance with the ARRIVE guidelines. The mice used in this study were obtained from the in-house breeding colony at the University of Basel Animal Facilities. The mice were housed and bred under a 12-h light/dark cycle at a controlled temperature and provided food and water *ad libitum*.

Both male and female animals were included in the experiments, with 3–5 females and 3–4 males used per genotype per experiment (6–9 animals per genotype in total). A total of 149 animals were used in this study, including 80 females and 69 males. Data from both sexes were pooled for analysis, and the potential influence of sex on the observed effects was not assessed separately. Organotypic cerebellar slice cultures were prepared under experimental license number 1708_38376 and as described previously^48^ from wild-type FVB/NRj, PKCγ-A24E^7^ or PKCγ-S361G^6^ mouse lines at postnatal day 8 or 9. Briefly, mice were decapitated and the brains were removed under aseptic conditions. The cerebellum was dissected in ice-cold Minimal Essential Medium (Sigma-Aldrich) supplemented with 1% Glutamax (Invitrogen), pH 7.3, and 350 μm thick sagittal sections were cut on a McIllwain tissue chopper. Slices were separated, transferred onto permeable membranes (Millicell-CM, Millipore), and incubated on a layer of Neurobasal medium (Invitrogen) containing 2% B27 supplement (Invitrogen) and 1% Glutamax (Invitrogen), in a humidified atmosphere with 5% CO2 at 37°C. The medium was refreshed every 2–3 days, and the slices were cultured for a total of 10 days.

### Method details

#### Reagents

The following chemical compounds were administered in the culture at each medium change to modify mGluR1 activity: 5 μM (RS)-3,5-dihydroxyphenylglycine (DHPG, Tocris Bioscience, 0342), 1 mM (S)-α-methyl-4-carboxyphenylglycine (MCPG, Tocris Bioscience, 0337). PKCγ-S361G mutant cultures were treated with 10 μg/mL tetracycline hydrochloride (tetracycline, PanReac AppliChem, A2228), referred to as PKC-S361G wild-type.

#### DNA constructs and adeno-associated viral (AAV) transduction

The pAAV/L7-6-GFP-WPRE plasmid^47^ was a gift from Hirokazu Hirai (Addgene plasmid # 126462). The vector was modified to reduce length and introduce additional restriction sites using the following primers: 5′-TTATGCTAGCTTCTAGAGCATGGCTACGT-3′ and 5′-ATAAACGCGTCTCGAGTCTAGAGCATGGC-3'. The pAAV/L7-6-GFP-WPRE-PKC-A24E plasmid was generated by amplifying the PKC-A24E insert^7^ with primers 5′-CGTGAGGACTACCGGTATGGCTGGTCTGGGCCCC-3′, and 5′-CATGGTGGCGTCGACCATGACGGGCACAGGCACTGG-3′, using CloneAmp HiFi PCR Premix (Takara Bio, 639298) according to the manufacturer’s instruction. The amplified fragment was inserted into the modified vector using the In-Fusion Snap Assembly Master Kit (Takara Bio, 638947). Plasmids were transformed into Stellar competent cells (Takara Bio, 636763), and colonies were selected on LB agar containing 100 μg/mL ampicillin. Plasmid integrity was verified by sequencing (Microsynth, Switzerland). AAVs were produced by PackGene Biotech AG. Organotypic slice cultures were infected at DIV0 with 0.75 × 10^11^ GC/mL of either pAAV/L7-6-GFP-WPRE or pAAV/L7-6-GFP-WPRE-PKC-A24E.

#### Protein extraction and western blot

Proteins were extracted from organotypic cerebellar slice cultures at DIV10 in lysis buffer (50 mM Tris-HCl, 0.15 M NaCl, 0.5% sodium deoxycholate, 1% NP-40, 0.1% SDS, 1 mM EDTA, 10 mM sodium fluoride, pH 7.4) supplemented with protease inhibitors (cOmplete, Sigma-Aldrich, 11873580001) and phosphatase inhibitors (PhosSTOP EASYpack, Roche, 04906837001). Samples were homogenized in Lysing Matrix D Tubes (MP Biomedicals, 6913100) using a FastPrep 24 5G homogenizer (MP Biomedicals) at 6 m/s for 40 s. Homogenates were clarified by centrifugation at 18,000 × g for 15 min at 4°C, and supernatants were collected and stored at −80°C. Protein concentrations were determined using the BCA protein assay kit (Merck Millipore, 71285).

Samples were mixed with 2× Laemmli buffer (Sigma-Aldrich, S3401) prior to loading onto 8% polyacrylamide gels. Gels were run at 30 mA (200 V) for 1 h in running buffer (0.25 M Tris, 1.93 M glycine, 0.1% SDS) using a Bio-Rad system. Proteins were then transferred to nitrocellulose membranes (Bio-Rad, 1620115) in transfer buffer (0.25 M Tris, 1.93 M glycine, 20% methanol) at 360 mA (250 V) for 1 h. Membranes were blocked with 5% bovine serum albumin (BSA, Sigma-Aldrich, A9647) in Tris-buffered saline (TBS, 0.05 M Tris, 0.15 M NaCl, pH 7.4) for 1 h at room temperature. Primary antibodies were applied in 5% BSA-TBS overnight at 4°C, as follows: mouse monoclonal anti-β-actin (1:2000, Sigma-Aldrich, A5441), rabbit polyclonal anti-phospho-serine (pSer) PKC substrate (1:1000, Cell Signaling Technology, 2261), mouse monoclonal anti-PKC Gamma (1:1000, Proteintech, 66429-1-Ig). Membranes were washed three times with TBS-0.1% Tween 20 (TBST) and incubated with secondary antibodies in TBST for 2 h at room temperature: donkey anti-mouse IRDye 800CW (1:10000, LI-COR Biosciences, 926–32212), donkey anti-rabbit IRDye 680LT (1:10000, LI-COR Biosciences, 926–68023). Signals were detected using the LI-COR Odyssey imaging system (LI-COR Biosciences). Average intensity values were calculated using Image Studio Lite 5.2 software (LI-COR Biosciences).

#### Immunohistochemistry

At 10 days *in vitro*, slices were fixed in 4% paraformaldehyde for 6 h at 4°C. After fixation, slices were washed in 100 mM phosphate buffer saline (PBS), pH 7.3, and incubated in PBS containing 0.1% Triton X-100 and 3% normal goat serum for 2 h at room temperature. Subsequently, the corresponding primary antibody mouse monoclonal anti-Calbindin (1:1000, Swant, CB300), guinea pig polyclonal anti-Calbindin (1:1000, Synaptic Systems, 214004), mouse monoclonal anti-PKC Gamma (1:200, Thermo Fisher Scientific, 13–3800), guinea pig polyclonal anti-Shank2 (1:1000, Synaptic Systems, 162204), mouse monoclonal anti-mGluR1 (1:500, BD Biosciences, 556331), rabbit monoclonal anti-mGluR1 (1:500, Cell Signaling Technology, 12551), rabbit polyclonal anti-Homer3 (1:500, Synaptic Systems, 160303), mouse monoclonal anti-GluR2 (1:100, Synaptic Systems, 182411) was added to the slices in PBS containing 0.1% Triton X-100 and 3% normal goat serum and incubated overnight at 4°C. After washing in PBS, appropriate secondary antibodies goat anti-mouse Alexa Fluor 488 (1:500, Thermo Fisher Scientific, A11001), goat anti-rabbit Alexa Fluor 568 (1:500, Thermo Fisher Scientific, A11011), goat anti-mouse Alexa Fluor 647 (1:500, Abcam, ab150115), goat anti-guinea pig Alexa Fluor 488 (1:500, Thermo Fisher Scientific, A11073), goat anti-guinea pig Alexa Fluor 647 (1:500, Thermo Fisher Scientific, A21450) were added to the slices in PBS containing 0.1% Triton X-100 for 2 h at room temperature. Slices were mounted on glass slides with Mowiol (Sigma-Aldrich) or ProLong Gold Antifade Mountant (Thermo Fisher Scientific, P36930) for subsequent microscopy analysis.

#### Image acquisition and quantitative analysis

Analysis of dendrites and dendritic spines in Purkinje cells was conducted using Calbindin staining, a calcium-binding protein exclusively expressed in Purkinje cells within the cerebellum.^49^

For dendrite analysis, images were acquired using an Olympus AX-70 microscope with a 20× objective. Dendritic arbor measurements were performed in Fiji using the AnalyzeSkeleton plugin,^50^ and Sholl analysis.^51^

Images for dendritic spine analysis were captured using a Nikon AX R with NSPARC detector (100×/1.45 NA oil immersion objective) at the Department of Biomedicine (DBM) Microscopy Core Facility. Z-stacks were acquired with 0.12 μm step size to ensure complete dendrite coverage and full field images of 2048 pixels (pixel size = 0.05 μm) were obtained. Manual investigation was performed on well-resolved dendritic segments, and analysis was limited to spines clearly visible in maximum intensity projections of z stack images. Spine types of distal dendritic branches were distinguished according to previously established criteria.^52^ Dendritic spines were classified by manually measuring the length (L), head width (H), and neck width (N) of each individual protrusion using the Fiji plugin, Dendritic Spine Counter. Spines were categorized quantitatively into mushroom, stubby, or thin types based on two ratios: the head-to-neck diameter ratio (H/N), which is greater than 1 for mushroom spines due to their characteristic bulbous head and narrow neck, and the length-to-neck ratio (L/N), used to differentiate short stubby spines from long thin spines.^53^ Specifically, mushroom spines were defined as those with an H/N ≥ 1.5; stubby spines had both H/N ≤ 1 and L/N ≤ 1; thin spines had H/N values between 1 and 1.5 and L/N values between 1.5 and 3. Filopodia were defined as spines with an H/N < 1.2 and an L/N > 3. Spines that did not meet any of these criteria were classified as unclassified.^19^

Synapse quantification and colocalization analyses were performed using the same microscope and acquisition settings as described for dendritic spine imaging. In all parallel experiments, images were acquired using identical acquisition parameters. For image analysis, dendritic branches were manually outlined, and the intensity of mGluR1, PKCγ and GluR2 and the number and size of Shank2-and Homer3-positive puncta within dendritic segments were quantified with a custom Fiji macro.^54^ Colocalization analysis was performed using the JACoP (Just Another Colocalization Plugin) plugin in Fiji,^55^ and Pearson correlation coefficient was calculated based on Costes automatic thresholding.

### Quantification and statistical analysis

#### Statistical analysis

Statistical analysis was computed by using GraphPad Prism 10. Normality of the data was calculated using the Shapiro-Wilk or Kolmogorov-Smirnov test. Depending on the normality of the data, comparisons between groups were made using either one-way ANOVA with Bonferroni correction or Welch and Brown-Forsythe ANOVA followed by Games-Howell or Dunnett post hoc test or Kruskal-Wallis test followed by Dunn’s multiple comparison test. A significance level of 0.05 was used for all statistical tests. The details of the specific test used in each case are provided in the figure legends.
